# Complementation of the *Mycoplasma synoviae* MS-H vaccine strain with wild-type *obg* influencing its growth characteristics

**DOI:** 10.1371/journal.pone.0194528

**Published:** 2018-03-28

**Authors:** Muhammad A. Shahid, Marc S. Marenda, Philip F. Markham, Amir H. Noormohammadi

**Affiliations:** 1 Faculty of Veterinary and Agricultural Sciences, The University of Melbourne, Werribee, Victoria, Australia; 2 Faculty of Veterinary and Agricultural Sciences, The University of Melbourne, Parkville, Victoria, Australia; Instituto Butantan, BRAZIL

## Abstract

The temperature-sensitive (*ts*^+^) *Mycoplasma synoviae* vaccine strain MS-H harbors a non-synonymous mutation which results in Glycine to Arginine substitution at position 123 in the highly conserved glycine-rich motif of Obg-fold in the GTP-binding protein Obg. *In-silico* analysis of the wild-type and mutant Obgs of *M*. *synoviae* has indicated that this amino acid substitution affects structure of the protein, potentially leading to abrogation of Obg function *in vivo*. Present study was conducted to develop the first expression vector for *M*. *synoviae* and to investigate the potential effect(s) of complementation of MS-H vaccine with the wild-type *obg* from 86079/7NS, the parent strain of MS-H. An *oriC* vector, pKS-VOTL, harboring the 86079/7NS *obg* gene, downstream of the variable lipoprotein haemagglutinin (*vlhA*) gene promoter, also cloned from 86079/7NS, was used to transform MS-H. The plasmid was localised at the chromosomal *oriC* locus of MS-H without any detectable integration at the chromosomal *obg* locus. Analysis of the MS-H transformants revealed abundant *obg* transcripts as well as Obg protein, when compared to the MS-H transformed with a similar vector, pMAS-LoriC, lacking *obg* coding sequence. The MS-H transformants complemented with wild-type Obg maintained their original temperature-sensitivity phenotype (consistent with MS-H vaccine) but, when compared to the MS-H transformed with pMAS-LoriC, had significantly higher (p < 0.05) growth rate and viability at the permissive (33°C) and non-permissive temperature (39.5°C), respectively. Analysis of Obg expression in MS-H and its wild-type parent strain revealed comparatively lower levels of Obg in MS-H. These results indicate that not only the mutation in Obg, but also the level of Obg expression, can confer functional abnormalities in the bacterial host. Furthermore, with the construction of first expression vector for *M*. *synoviae*, this study has set foundation for the development of recombinant vaccine(s) based on MS-H.

## Introduction

*Spo**0B*-associated GTP-binding protein (Obg) belongs to OBG-HflX superfamily within the TRAFAC (translational factors) class of P-loop (phosphate-binding loop) GTPases which are found in all living organisms ranging from human to bacteria [1–3]. Originally identified in *Bacillus subtilis* [4], other members of Obg subfamily include Obg from *Streptomyces coelicolor* and *Streptomyces griseus*, CgtA from *Caulobacter crescentus*, *Escherichia coli* (*E*. *coli* CgtA is also called ObgE), *Vibrio harveyi* and YhbZ from *Haemophilus influenzae* [5]. Obg GTPases are involved in essential cellular functions including cell growth, morphological differentiation, DNA replication [6], chromosome segregation [7], early steps of sporulation [8], ribosome assembly [9] and stress dependent activation of σ^B^ transcription factor [10, 11].

Temperature-sensitive (*ts*) mutants of *obg* have been isolated and studied in different bacteria especially *B*. *subtilis* and *E*. *coli* [12]. A mutation G79E in the type-II helix d and D84N in the N-terminal Obg domain was shown to be the contributing factor of temperature sensitivity in *B*. *subtilis* [6, 13]. Corresponding mutations in *E*.*coli* ObgE, G80E and D85N, also resulted in no growth at non-permissive temperature of 42°C [14]. ObgEts mutant of another *E*. *coli* strain with point mutation (S314P) in GTPase G5 motif exhibited growth defects at elevated temperature [14, 15]. Such observations suggest crucial roles for the N-terminal domain of Obg proteins. The mechanistic insight into how Obg participates in diverse cellular functions is yet to be deciphered but bacterial Obg proteins have been found associated with ribosome(s) and therefore appear to be involved in ribosome biogenesis, maturation and assembly [15–21]. Consequently, Obg proteins are considered essential for cell viability in various bacteria [3] including *Mycoplasma genitalium* [22, 23].

The *Mycoplasma synoviae* vaccine MS-H is a *ts*^*+*^ strain which is used to control *M*. *synoviae* infections in poultry. In our previous study, analysis of single nucleotide polymorphisms (SNPs) in *obg* gene of MS-H highlighted the putative role of Obg in temperature sensitivity and/or attenuation of MS-H [24]. However molecular basis of the attenuation of MS-H vaccine has not been fully investigated under experimental conditions so far. In fact, failure to understand the mechanism of temperature sensitivity and/or attenuation of MS-H vaccine, and mechanism of *M*. *synoviae* pathogenesis in general, is partly due to lack of genetic tools to manipulate this organism at molecular levels. Development of gene transfer methods, as described in our previous study [25], has made it possible to study the role of putative virulence factors in *M*. *synoviae* pathogenesis and to underpin the molecular basis of MS-H temperature sensitivity and/or attenuation. This study describes, for the first time, the cloning and expression of a foreign gene (*obg*) in *M*. *synoviae*, and further evaluates the effect of MS-H complementation with wild-type *obg* on its temperature-sensitivity and/or growth characteristics.

## Materials and methods

### Ethics statement

Fourteen-day old specified pathogen free chickens (n = 3) were used in this study. Throughout the experiment, chickens were housed in high-efficiency particulate air-filtered negative pressure isolators within animal house facility of Asia Pacific Centre for Animal Health, Faculty of Veterinary and Agricultural Sciences, The University of Melbourne, Werribee, Victoria, Australia. Chickens were provided irradiated food and acidified water *ad libitum*. Purified recombinant Obg-MBP protein, emulsified with Montanide ISA 71 VG (Seppic Inc., Puteaux, France) adjuvant, was used to immunise chickens by intramuscular injection. Similarly, two booster injections were also administered and blood samples of approximately 1 ml were taken from jugular vein after 14 days following each injection. At termination of the experiment, birds were bled (maximum 5% of the body weight) from jugular vein with a 10 ml syringe and 21-gauge needle and then euthanized using intravenous injection of phenobarbitone. The University of Melbourne Animal Ethics Committee specifically approved this whole study (approval number 0911553.1).

### Bacterial strains and culture conditions

*M*. *synoviae* strains/transformants, listed in Table 1, were grown to late exponential phase (~ pH 6.8) at 37°C, unless otherwise stated, in mycoplasma broth (MB) as described previously [26], except MB and mycoplasma agar (MA) contained 3 μg mL –^1^ tetracycline (Sigma, St. Louis, Missouri, USA) for the growth of tetracycline resistant *M*. *synoviae* strains transformed with pMAS-LoriC and pKS-VOTL plasmids. *Escherichia coli* α-Select (DH5 α derivative) competent cells (Bioline, Alexandria, New South Wales, Australia) were used as host for gene cloning and propagation of different plasmids following manufacturer’s instructions.

**Table 1:** *M*. *synoviae* strains and plasmids used in this study.

*M*. *synoviae* strains or plasmids	Relevant genotype/characteristics	Reference
*M. synoviae* strains		
MS-H	*M. synoviae* vaccine strain (ts^+^)	[27]
86079/7NS	MS-H parent strain (*ts*^–^)	[28]
MS-H5	*ts*^−^reisolate of MS-H	[24, 29]
93198/1-24b	*ts*^−^reisolate of MS-H	[24, 29]
MS-H-C28	MS-H transformed with pMAS-LoriC	[25]
MS-H-T75, MS-H-T78, MS-H-T90	MS-H transformed with pKS-VOTL	This study
7NS-C38, 7NS-C12	86079/7NS transformed with pMAS-LoriC	This study
7NS-T30, 7NS-T20, 7NS-T8, 7NS-T4	86079/7NS transformed with pKS-VOTL	This study
Plasmids		
pMAS-LoriC	LoriC-pGEM-T *tet*	[25]
pKS-VOTL	LoriC-pBluescript II KS (+) *tet* P_vlhA_- *obg*^wt^	This study
pGEM-T	Cloning vehicle	Promega
pBluescript II KS (+)	Cloning vehicle	Thermo Fisher Scientific
pMAL-p2	Expression vector	New England Biolabs
pMAL-obg	pMAL-p2 Δ*obg*	This study

### Construction of the *vlhA*-*obg*-*oriC* plasmid (pKS-VOTL)

**Fig 1:**
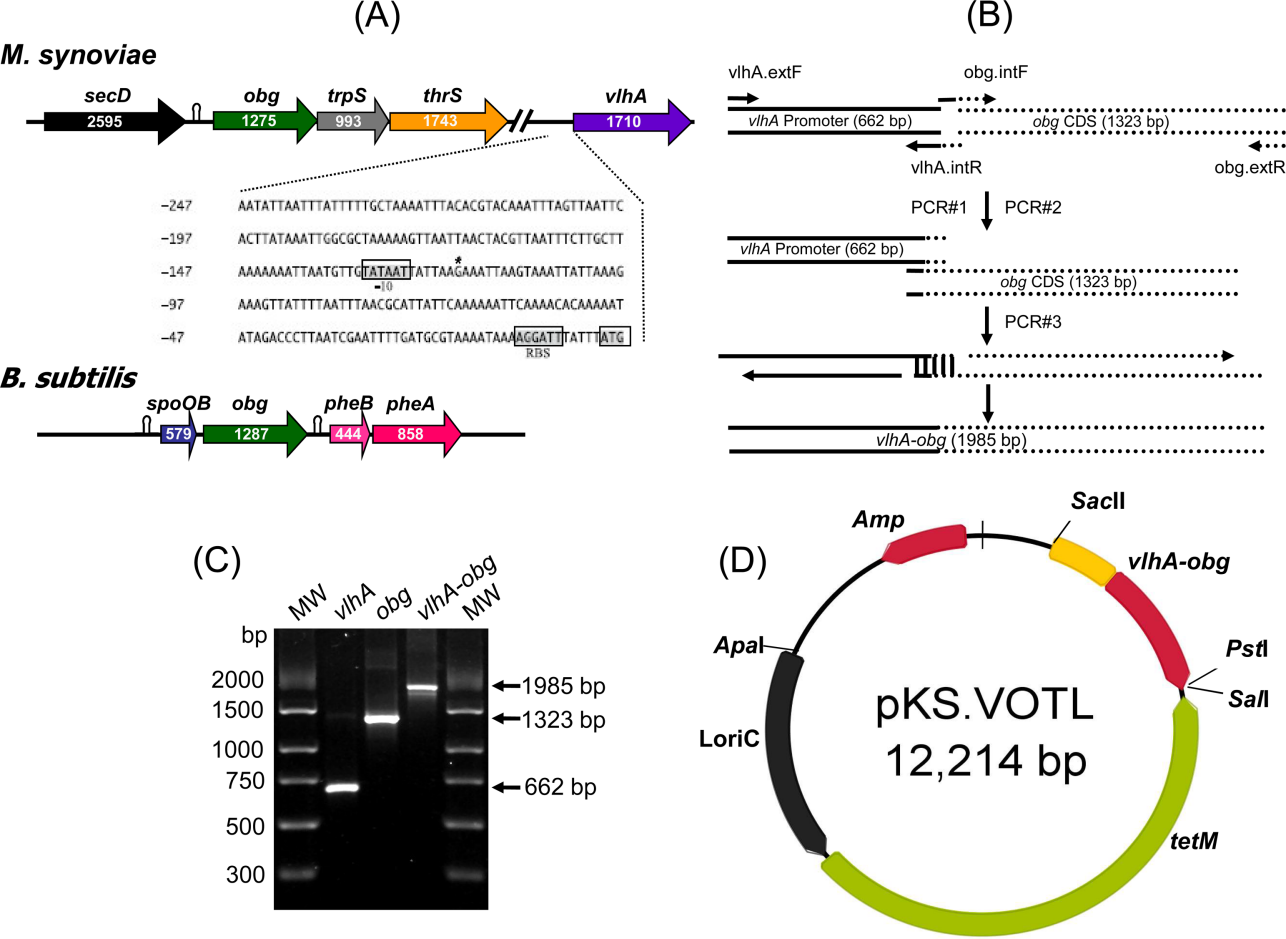
Construction of pKS-VOTL plasmid. (A) Organisation of *spo0B* operon in *B*. *subtilis* and putative *obg* operon in *M*. *synoviae* has been shown. Putative –10 promoter region, transcription start site, ribosome binding site (RBS) of *vlhA* promoter region, and initiation codon for *vlhA* gene have been indicated. Length (bp) of each CDS is indicated inside the arrows. Identified stem loop structures in *B*. *subtilis spo0B* operon and putative stem loop in *M*. *synoviae* ‘*obg* operon’ have also been indicated. (B) Schematic presentation of splicing by overlap extension (SOE) PCR to join *vlhA* gene promoter with *obg* CDS. Using PCR#1 and PCR#2, *vlhA* promoter (solid lines) and *obg* CDS (dotted lines) were amplified using indicated primers. Intermediate products with overlapping fragments (shown by horizontal bars) from both PCRs were amplified by SOE-PCR (PCR#3) using primers vlhA-ExtF and obg-ExtR. (C) Agarose gel electrophoresis of amplification products of *vlhA* promoter region PCR, *obg* CDS PCR and SOE-PCR. MW, DNA molecular weight marker (PCR Marker, Sigma, Missouri, USA). (D) Final product of SOE-PCR was first cloned at T-site of pGEM-T Easy Vector and then *Pst*I-*Sac*II fragment containing *vlhA*-*obg* was inserted between *Pst*I and *Sac*II sites of pBluescript II KS (+) vector. *Apa*I-*Sal*I restriction fragment containing LoriC and *tetM*, from pMAS-LoriC plasmid, was cloned at respective sites in pBluescript II KS (+) vector, harboring *vlhA*-*obg*, to generate pKS-VOTL plasmid.

In *B*. *subtilis*, *obg* is part of an operon consisting of an upstream gene *spo0B* and two downstream genes *pheB* and *pheA* (Kok et al., 1994). In *M*. *synoviae*, *obg* may also be part of a large operon consisting of upstream *secD* and downstream *trpS* and *thrS* genes. As *obg* gene regulatory elements are not known in *M*. *synoviae*, *vlhA* gene promoter region was selected to drive *obg* gene expression (Fig 1A) and *vlhA* gene promoter region was joined to *obg* CDS using splicing by overlap extension (SOE) PCR as described before [30] with modifications (S1 File). Amplification of *vlhA* promoter region was conducted using vlhA-extF and vlhA-intR primers in PCR#1 and *obg* CDS was amplified using obg-intF and obg-extR primers in PCR#2 (Fig 1B; S1 Table). Overlapping nucleotide sequences from these two PCRs were extended in the overlap extension PCR (PCR#3) using the oligonuclotides vlhA-extF and obg-extR (Fig 1B; S1 Table). Amplification conditions for the above 3 PCRs are described in S1 File. Amplicons of expected size were confirmed by agarose gel electrophoresis (Fig 1C) and *vlhA*-*obg* amplicon was cloned into pGEM-T Easy vector according to the manufacturer’s instructions (Promega, Alexandria, New South Wales, Australia). A *Pst*I-*Sac*II fragment containing *vlhA*-*obg* was excised and inserted into the compatible sites of pBluescript II KS (+) phagemid (Thermo Fisher Scientific, Scoresby, Victoria, Australia). The *Apa*I and *Sal*I restriction fragment, from pMAS-LoriC plasmid [25], containing LoriC (*M*. *synoviae* origin of replication) and *tetM* resistance marker was ligated into compatible sites of pBluescript II KS (+) phagemid already containing *vlhA*-*obg*. The final plasmid construct, designated as pKS-VOTL (for *v**lhA*, *o**bg*, *t**etM* and LoriC), was sequenced (BDT Version 3.1; Applied Biosystems, Foster City, California, USA) using suitable primers (S1 Table) to confirm inserts (*vlhA*-*obg*, LoriC-*tetM*) cloned into pKS-VOTL (Fig 1D). PureYield Plasmid Midiprep (Promega) of pKS-VOTL (~ 500 ng μL –^1^) was used to transform *M*. *synoviae* strain MS-H as described previously for pMAS-LoriC [25]. MS-H vaccine parent strain, 86079/7NS, was also transformed with pMAS-LoriC and pKS-VOTL, and resulting transformants, at *in vitro* passage number 8, were used as control only in Obg expression (Western immunoblotting) and growth comparison studies.

### Southern blot hybridisation

Genomic DNA from untransformed and transformed MS-H was extracted using Chen and Kuo [31] method as modified and described by Shahid et al. [25]. Genomic DNA from pKS-VOTL MS-H transformants, untransformed MS-H and pKS-VOTL plasmid was digested with restriction enzyme *Bgl*II (New England Biolabs, Wilbury Way, Hitchin, England) according to manufacturer’s recommendations. Digoxigenin (DIG)-labelled probes for *tetM*, *oriC* and *vlhA*-*obg* were synthesised using the Roche PCR-based method [32] by incorporating 0.5 μL of 1 mM DIG-11-dUTP (Roche Diagnostics, Mannheim, Baden-Württemberg, Germany) per PCR reaction. Blotting of the restriction fragments, separated on 1% agarose gel, onto positively charged Nylon membrane, hybridisation to DIG-labelled probes for *tetM*, *oriC* and *vlhA*-*obg*, and detection of chemiluminescent signals, was achieved as described previously [25].

### Northern blot hybridisation

Cell pellets from approximately 50 mL *M*. *synoviae* cultures were obtained by centrifugation at12,000×g for 40 min at 4°C and then immediately processed for total RNA extraction using TriPure reagent (Roche) as per manufacturer's recommendations. Concentration of RNA as determined by spectrophotometry (NanoDrop 1000; Thermo Fisher Scientific, Wilmington, Delaware, USA) at A260nm was adjusted to ~ 1μg μL –^1^ and then stored at –70°C until further use. RNA was mixed (1:1) with RNA loading buffer and then separated on 1% agarose gel, made in MOPS (4-morpholine propanesulfonic acid) buffer containing 1.85% formaldehyde (Sigma), using 1× MOPS (20 mM MOPS, 5 mM Na-Ac, 1 mM EDTA, pH 7.0) as running buffer. RNA gel was soaked in 20× SSC buffer to remove formaldehyde. DIG labelling of the probes (*obg* and *vlhA*) was performed as described above. Blotting of RNA onto positively charged Nylon membrane, hybridisation of DIG-labelled *vlhA*-*obg*, *obg* and *vlhA* probes (S1 Table) and detection of hybridised probes was performed as described for Southern blot hybridisation [25]. Northern blotting was performed in two separate experiments using approximately 25 and 50 μg total RNA per lane. Each blot was probed with three different probes as described above and a qualitative analysis was performed to determine if *obg* is transcribed under *vlhA* promoter or not.

### Production of chicken and rabbit polyclonal antibodies to *M*. *synoviae* Obg

Recombinant *M*. *synoviae* Obg was expressed in, and purified from, *E*. *coli* as Obg-MBP and used to inoculate chickens for polyclonal anti-Obg-MBP chicken serum (S2 File). Using GenScript OptimumAntigen design tool, several highly hydrophilic/antigenic epitopes were predicted in *M*. *synoviae* Obg. Based on criteria for surface probability, homology to mouse or rabbit proteins, flexibility, secondary structures, signal peptide and solubility, four peptides (Obg_1 TSHLQIKIEDDFET, Obg_2 GKGGRGNNKFKTSK, Obg_3 NTAPRIAENGMPGE and Obg_7 KMNRNHFKVTGKKI) were selected and synthesized (GenScript, Piscataway, New Jersey, USA). New Zealand rabbits were immunized with each of these peptides and affinity-purified polyclonal antibodies were produced (GenScript) and used in immunoblotting as described below.

### SDS-PAGE and Western immunoblotting

Whole cell proteins from *M*. *synoviae* cultures were subjected to SDS-PAGE and gels stained with Coomassie brilliant blue R-250 (Bio-Rad, Gladesville, New South Wales, Australia). For Western immunoblotting, unstained gels were used for protein transfer onto PVDF membrane using Trans-Blot Turbo RTA Mini PVDF Transfer Kit (Bio-Rad) using Trans-Blot Turbo Transfer System (Bio-Rad). Blotting membrane was blocked with 10% BSA overnight at 4°C, and then incubated with either polyclonal chicken anti-Obg-MBP serum or rabbit polyclonal anti-Obg peptide primary antibodies for 1 h at room temperature. After washing three times in PBS-T (PBS containing 0.1% Tween-20), the blot was incubated for 1 h with either rabbit anti-chicken or goat anti-rabbit IgG HRP conjugate (Millipore, Temecula, California, USA) (1:1000 dilution). The blot was developed with a solution of SIGMA*FAST* 3–3' Diaminobenzidine and Urea Hydrogen Peroxide tablets (Sigma) dissolved in distilled water. For comparative analysis of Obg expression, whole cell proteins from *M*. *synoviae* strains/clones MS-H, MS-H-C28, MS-H-T75, 86079/7NS, 7NS-C38 and 7NS-T30 were quantified using 2-D quant kit (GE Healthcare, Piscataway, NJ, USA) as per manufacturer's recommendations. Loading volumes for each sample were adjusted, using 2-D quant kit estimated whole cell protein concentrations, in order to load approximately equal amounts of whole cell proteins onto the SDS-PAGE gels and subsequently subjected to Western immunoblotting. Immunoblots were scanned using CanoScan 8800F (Canon, Sydney, NSW, Australia). Intensity of Obg specific bands was quantified with ImageJ 1.50i (Wayne Rasband, NIH, USA), using volume tool (rectangle) and local background subtraction, and relative concentration (fold change) estimated from at least three independent immunoblotting experiments.

### Temperature-sensitive phenotyping, growth curves and loss of viability study of MS-H and 86079/7NS transformants

Temperature-sensitive phenotype of MS-H, MS-H-C28, MS-H-T75, 86079/7NS, 7NS-C38 and 7NS-T30 was determined by *vlhA* Q-PCR and conventional microtitration as described previously [29]. For growth rate comparisons, transformant cultures, at an initial titre of ~10^7^ CCU mL –^1^, were diluted (1:1000) in MB containing 3 μg mL –^1^ tetracycline, and grown in triplicates (40 mL each) at 33°C for 192 h. Samples were taken in 24 h intervals and subjected to titration using titration plates incubated at 33°C for two weeks. Colour-changing units per ml (CCU mL –^1^) were calculated as described before [33]. Generation times, during the exponential phase of cultures, were calculated from the log_10_-transformed CCU mL –^1^ values plotted against time using the formula provided by Microbiology Laboratories (http://inst.bact.wisc.edu/inst/index.php; dated 26.2.2015):

k=(log10[Xt]−log10[X0])/(0.301×t)

where *k* is the growth rate (generations per hour), *Xt* is higher CCU mL –^1^, *X0* is lower CCU mL –^1^ and *t* is the time interval between *Xt* and *X0* (in hours). Generation time is reciprocal of *k* i.e. 1/*k* (hours per generation).

Temperature shift experiment was conducted to compare loss of viability of transformant cultures as described previously [29], with some modifications adopted from Taschner *et al*. [34] and Addinall *et al*. [35]. Briefly, MS-H-C28, MS-H-T75, 7NS-C38 and 7NS-T30 cultures, all at an initial titre of ~10^7^ CCU mL^–1^, were diluted (1:10) in MB and grown in triplicates in 40 mL aliquots at 39.5°C for 72 h. A 0.5 mL aliquot was taken from each culture in 12 h intervals and subjected to titration in a microtitre plate incubated at 33°C for two weeks.

### Statistical analysis

For quantitative analysis of Western blots, intensities of Obg specific bands were averaged from at least three independent experiments and relative fold change was estimated for Obg protein. Growth curve and loss of viability data is expressed as mean ± standard deviation from three independent experiments. Using GraphPad Prism (version 5.01), log_10_-transformed CCU mL^–1^ values at each time interval, from three independent experiments, for each transformant were compared using Student's *t*-test. Differences with *p* values < 0.05 were considered statistically significant and indicated by "*".

## Results

### Integration of pKS-VOTL plasmid in *oriC* region of MS-H vaccine strain

**Fig 2:**
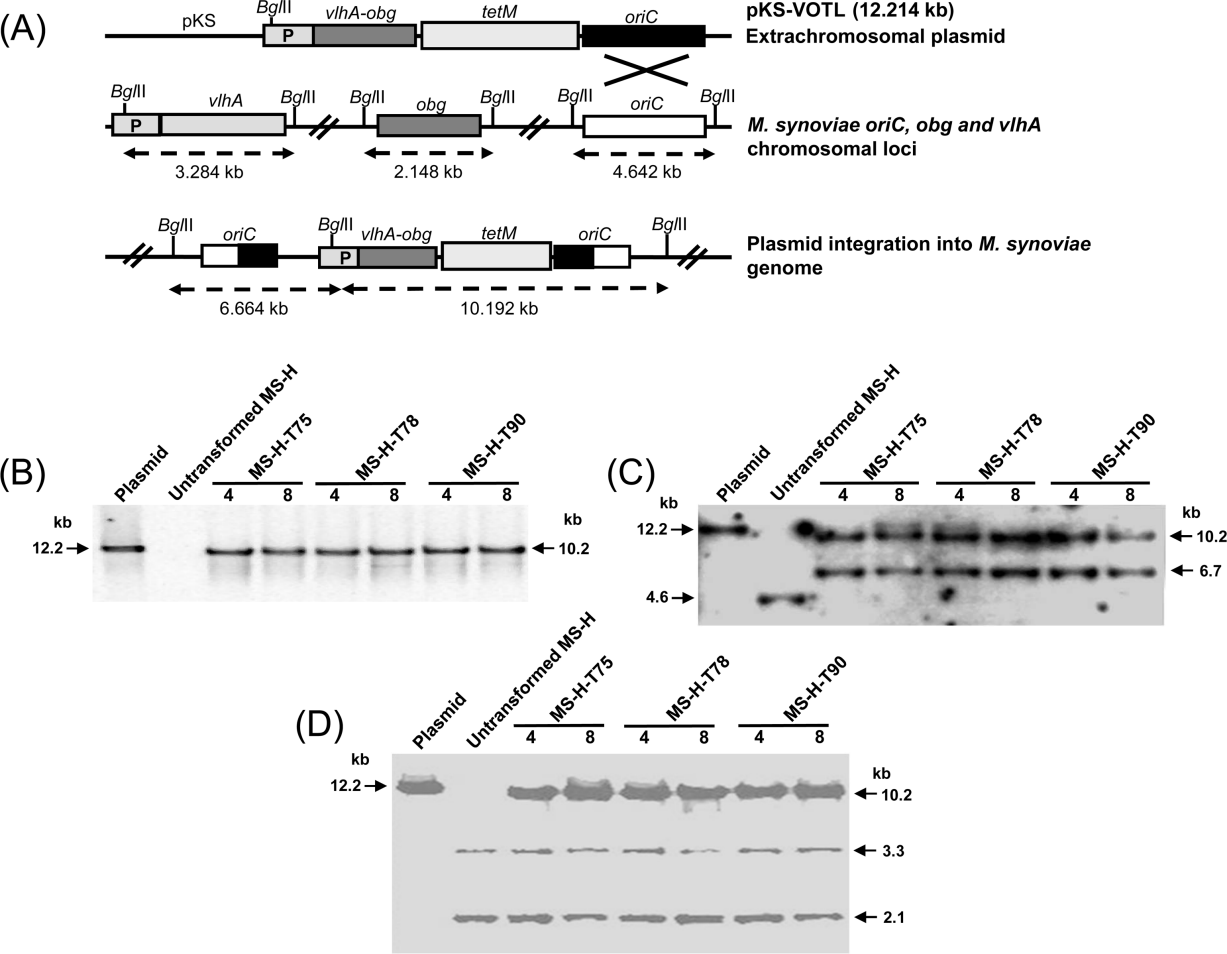
Southern blot hybridisation to localise pKS-VOTL plasmid in MS-H transformants. (A) Schematic presentation for the integration event of pKS-VOTL plasmid into genomic DNA of MS-H. A single putative homologous recombination event between the *oriC* copy carried by plasmid and chromosomal *oriC* region is represented by crossed lines. P indicates the promoter region of *vlhA* gene. (B, C, and D) *Bgl*II digested DNA of pKS-VOTL, untransformed MS-H and pKS-VOTL MS-H transformants MS-H-T75, MS-H-T78, MS-H-T90 (at passage 4^th^ and 8^th^) was hybridised with DIG labeled *tetM* (B), *oriC* (C) and *vlhA*-*obg* probes (D).

Following *Bgl*II digestion, genomic DNA of three MS-H transformant clones (MS-H-T75, MS-H-T78 and MS-H-T90) was subjected to Southern blot hybridisation to locate pKS-VOTL plasmid in MS-H cells. *Bgl*II digestion of unintegrated pKS-VOTL was expected to produce a single band of ~ 12.2 kb which was detected by DIG-labeled *tetM* probe (Fig 2A and 2B). In case of integration at the chromosomal *oriC* locus, two fragments of ~ 6.7 and 10.2 kb were expected to be produced, and only one band of ~ 10.2 kb hybridised to *tetM* probe as expected. Absence of ~ 12.2 kb band in all pKS-VOTL transformants, at both 4^th^ and 8^th^ passage level, indicated no extrachromosomal form of the pKS-VOTL existed in MS-H (Fig 2A and 2B). DIG-labeled *oriC* probe was expected to bind to a single *Bgl*II fragment of ~ 12.2 kb in case of free pKS-VOTL plasmid (Fig 2A and 2C). A single DNA fragment of 4.6 kb from untransformed MS-H, digested by *Bgl*II, hybridised to *oriC* probe as expected (Fig 2C). In case of pKS-VOTL transformants, following integration at *oriC* locus, two fragments of 6.7 and 10.2 kb hybridised to *oriC* probe as expected (Fig 2C). A DIG-labeled probe, *vlhA*-*obg*, spanning the joining site of *vlhA* promoter region with *obg* CDS, was also used to localise integration site of the plasmid. Only a ~ 10.2 kb band was detected by *vlhA*-*obg* probe, indicating absence of free pKS-VOTL plasmid in transformants. DIG-labeled *vlhA*-*obg* probe hybridised to bands of expected sizes (~ 2.1 and 3.3 kb for *obg* and *vlhA* loci, respectively), following *Bgl*II digestion of the genomic DNA from untransformed and transformed MS-H, indicating absence of homologous recombination event occurring in transformants at any other locus except chromosomal *oriC* (Fig 2A and 2D).

### Abundant *obg* transcripts produced under *vlhA* promoter in MS-H vaccine strain

**Fig 3:**
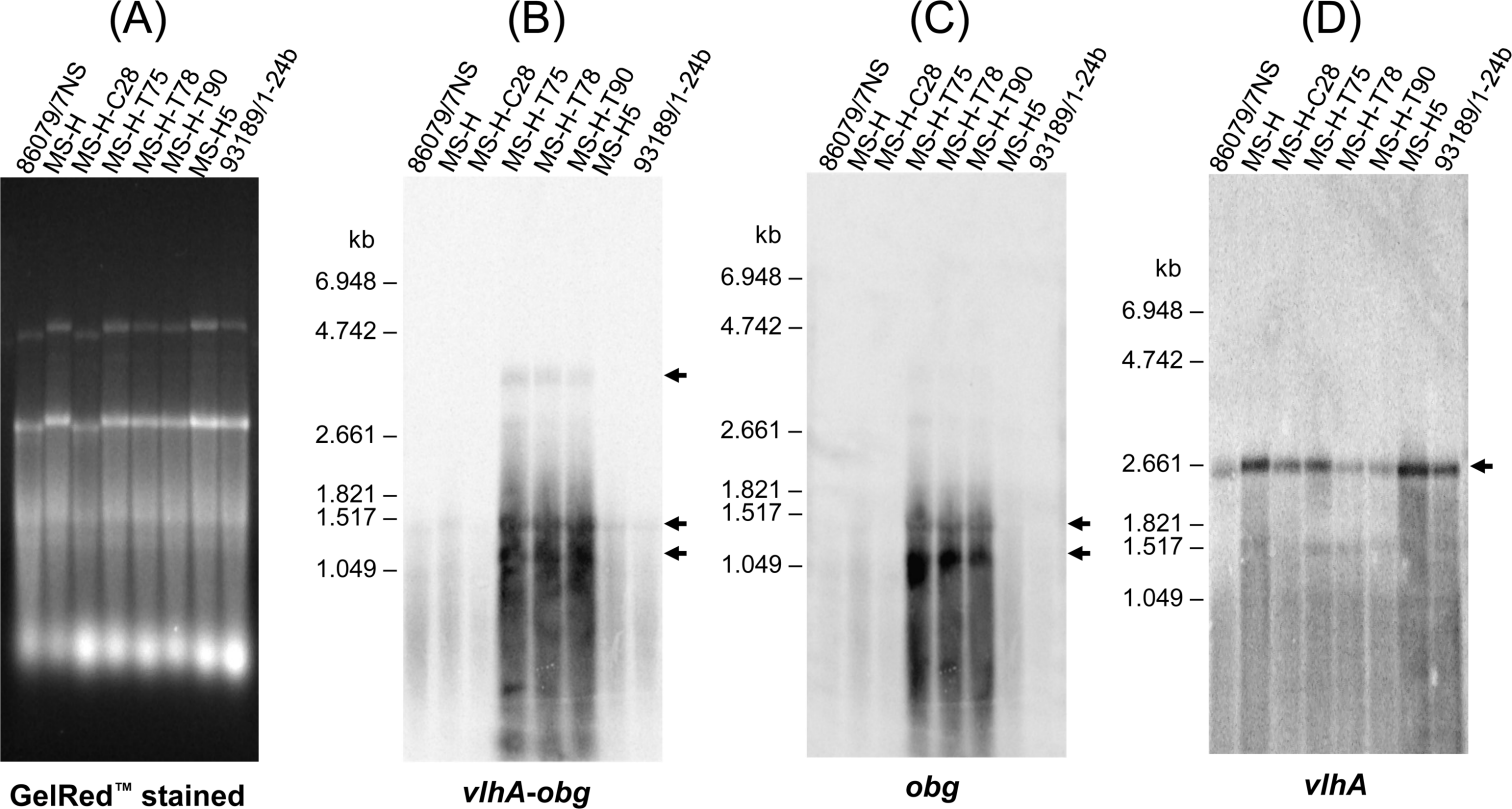
Northern analysis of total RNA (~ 50 μg/lane) from *M*. *synoviae* 86079/7NS, MS-H, MS-H-C28, MS-H-T75, MS-H-T78, MS-H-T90, MS-H5 and 93198/1-24b. (A) Agarose gel of total RNA from untransformed (86079/7NS, MS-H, MS-H5 and 93198/1-24b) and transformed (pMAS-LoriC transformant MS-H-C28 and pKS-VOTL transformants MS-H-T75, MS-H-T78 and MS-H-T90) *M*. *synoviae* MS-H stained with GelRed. (B, C, and D) Northern blot of the above gel hybridised with DIG-labeled *vlhA*-*obg* probe (spanning the joining site of *vlhA* promoter with *obg* CDS), *obg* specific probe and *vlhA* coding sequence specific probe, respectively. Arrowheads indicate the location of the specific bands identified by probes described above. The location of bands for the DIG-labeled RNA molecular weight marker RNA I (Roche) is indicated on the left side of all northern blots. ImageJ (1.48v; NIH, USA) was used to quantify intensity of *obg* transcripts.

Qualitative Northern blot hybridisation analysis was performed only to demonstrate if *obg* is transcribed or not under *vlhA* promoter in MS-H clones MS-H-T75, MS-H-T78 and MS-H-T90 (transformed with *obg*-containing pKS-VOTL). Integrity of the total RNA extracted from 86079/7NS, MS-H, MS-H-C28 (MS-H transformed with pMAS-LoriC), MS-H-T75, MS-H-T78, MS-H-T90 and two untransformed *ts*^*−*^reisolates of MS-H (MS-H5 and 93198/1-24b) was demonstrated by denaturing agarose gel stained with GelRed (Fig 3A). Slight fluctuation of rRNA bands in the left three samples was observed which might have been caused by different concentration of salts in the specimens loaded on each lane (Fig 3A). Northern blot analyses of approximately equal quantities (~ 50 μg per lane) were conducted. Hybridisation of the blot with *vlhA*-*obg* probe resulted in detectable transcripts of ~ 1.4 and/or 1.2 kb in all clones examined; however, intensity of both bands from three different MS-H pKS-VOTL transformants was noticeably higher than all other clones. A further transcript of ~ 3.9 kb was detected only in the three pKS-VOTL transformants of MS-H (Fig 3B). Hybridisation of the blot with the *obg* probe generated two bands of ~ 1.4 and ~ 1.2 kb in transformants MS-H-T75, MS-H-T78 and MS-H-T90. These bands were only barely detectable in Northern blots of other clones (Fig 3C), and almost undetectable in northern blots with lower quantity (~ 25 μg per lane) of total RNA (S1 Fig). The *vlhA* probe (specific for the conserved 5^′^ end of the highly expressible *vlhA* gene) detected a transcript of ~ 2.6 kb with variable intensity in all clones (Fig 3D). The *vlhA* probe was used only to differentiate *vlhA* transcripts from *obg* transcripts. These results indicated that the transcript of ~ 3.9 kb was transcribed from integrated plasmid harboring wild-type *obg* under *vlhA* promoter and transcripts of ~ 1.4 and/or 1.2 kb were most likely transcribed from endogenous *obg*.

### Overexpression of wild-type Obg in pKS-VOTL transformed MS-H and 86079/7NS

**Fig 4:**
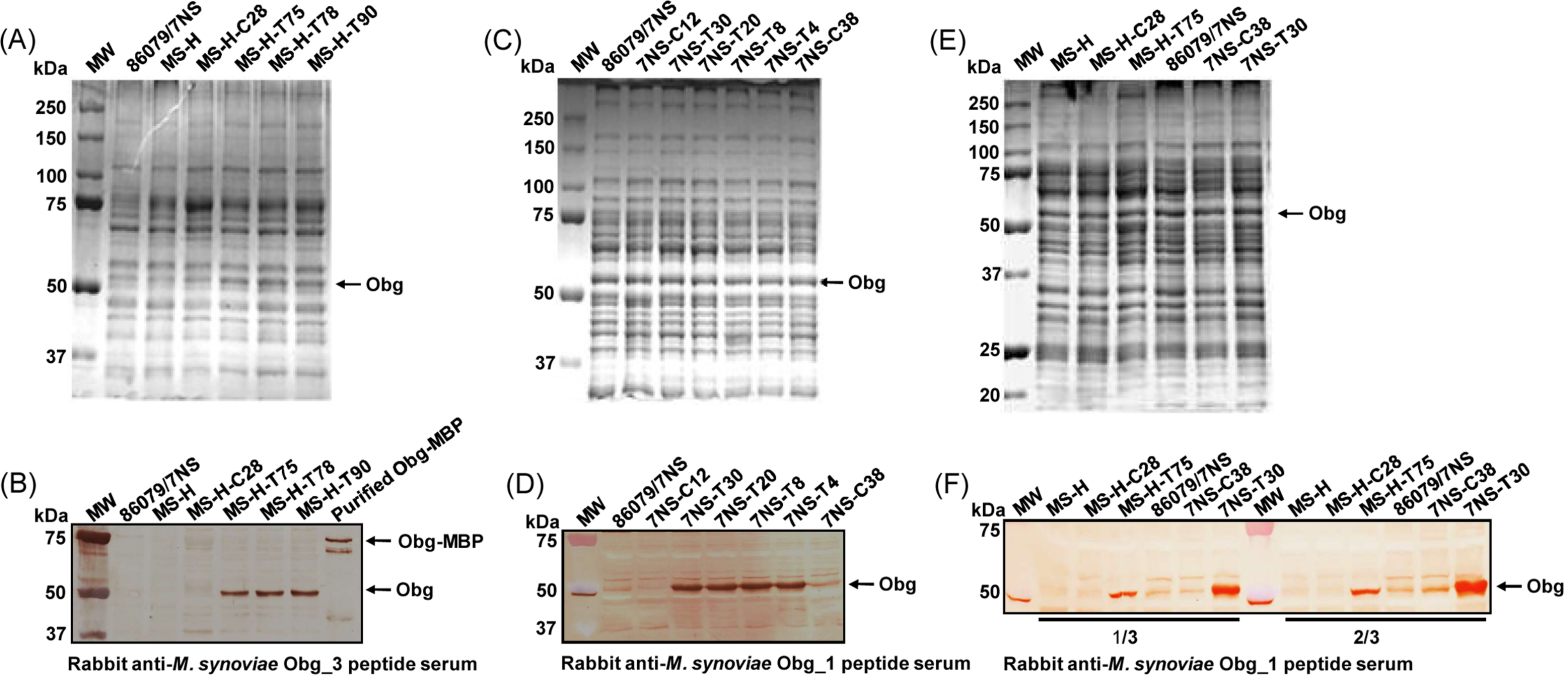
Analysis of Obg expression in *M*. *synoviae* strains using SDS-PAGE and immunoblotting. (A) SDS-PAGE (7.5%) of whole cell proteins from *M*. *synoviae* MS-H transformed with pMAS-LoriC (clone MS-H-C28) and pKS-VOTL (clones MS-H-T75, MS-H-T78 and MS-H-T90) plasmids, stained with Coomassie brilliant blue R-250 (Bio-Rad). Location of ~ 50 kDa protein in pKS-VOTL transformants is indicated with an arrow. (B) Immunostaining of untransformed 86079/7NS, MS-H, and transformed MS-H with pMAS-LoriC (clone MS-H-C28) and pKS-VOTL (clones MS-H-T75, MS-H-T78 and MS-H-T90) using polyclonal rabbit serum against *M*. *synoviae* Obg_3 peptide. Location of Obg-MBP fusion protein and overexpressed Obg is indicated with arrows on the right. (C) SDS-PAGE (8.75%) of whole cell proteins from *M*. *synoviae* 86079/7NS transformed with pMAS-LoriC (clone 7NS-C12 and 7NS-C38) and pKS-VOTL (clones 7NS-T30, 7NS-T20, 7NS-T8 and 7NS-T4) plasmids, stained with Coomassie brilliant blue R-250 (Bio-Rad). (D) Immunostaining of untransformed 86079/7NS and transformed 86079/7NS with pMAS-LoriC (clone 7NS-C12 and 7NS-C38) and pKS-VOTL (clones 7NS-T30, 7NS-T20, 7NS-8 and 7NS-T4) using polyclonal rabbit serum against *M*. *synoviae* Obg_1 peptide. Location of overexpressed Obg is indicated with an arrow on the right. (E) For comparison of Obg expression in MS-H and 86079/7NS, approximately equal amounts (88 μg/lane) of whole cell proteins from MS-H, MS-H-C28, MS-H-T75, 86079/7NS, 7NS-C38 and 7NS-T30 were separated by SDS-PAGE (8.75%) and stained with Coomassie Brilliant Blue. (F) Immunostaining at two different concentrations of whole cell proteins (i.e. 1/3 and 2/3 of that loaded onto SDS-PAGE as shown in panel E) of each strain/transformant using polyclonal rabbit serum against *M*. *synoviae* Obg_1 peptide. MW, protein marker (Precision Plus Protein, Dual Color, Bio-Rad).

Expression of wild-type Obg under *vlhA* promoter from MS-H and 86079/7NS transformants with pKS-VOTL was demonstrated by SDS-PAGE of whole cell proteins (Fig 4A and 4C). Polyclonal chicken serum raised against Obg-MBP could not detect a band of ~ 50 kDa in *M*. *synoviae* strains (S2 Fig). Polyclonal rabbit sera against *M*. *synoviae* Obg peptides Obg_1, Obg_2, Obg_3 and Obg_7 detected a band of ~ 50 kDa in pKS-VOTL transformants; however, polyclonal rabbit serum against Obg_1 and Obg_3 mainly reacted to ~ 50 kDa band (Fig 4B and 4D) whilst Obg_2 and Obg_7 reacted to several bands including one of ~ 50 kDa (results not shown). Specificity of the immunostaining for *M*. *synoviae* Obg was further confirmed by staining of a band of ~ 77 kDa corresponding to recombinant Obg-MBP (Fig 4B). Using Obg_1 polyclonal rabbit serum, the pKS-VOTL transformed 86079/7NS clones (7NS-T30, 7NS-T20, 7NS-T8 and 7NS-T4) exhibited strong reaction with ~ 50 kDa band whilst pMAS-LoriC transformants (7NS-C38 and 7NS-C12) and untransformed 86079/7NS exhibited weaker, but detectable, reaction with Obg_1 polyclonal rabbit serum (Fig 4D).

### Relatively lower expression levels of endogenous Obg in MS-H vaccine strain than 86079/7NS

At approximately equal concentrations of whole cell proteins, determined by 2D quant kit, from *M*. *synoviae* strains MS-H, MS-H-C28 (MS-H transformed with pMAS-LoriC), MS-H-T75 (MS-H transformed with pKS-VOTL), 86079/7NS, 7NS-C38 (86079/7NS transformed with pMAS-LoriC) and 7NS-T30 (86079/7NS transformed with pKS-VOTL) (Fig 4E), the MS-H and MS-H-C28 showed no detectable reaction with the Obg_1 polyclonal rabbit serum at two different concentrations examined, whilst 86079/7NS and 7NS-C38 showed detectable reaction which increased on doubling the protein concentration (Fig 4F and S3 Fig). The reaction of 7NS-T30 with rabbit polyclonal anti-Obg peptide primary antibodies was also higher, ~ 2.7 ± 0.5-fold with Obg_1 (n = 5), than that of MS-H-T75 (Fig 4F and S3 Fig). MS-H5 (*ts*^*−*^MS-H reisolate) also exhibited detectable levels of Obg at the whole cell protein concentrations examined when probed with rabbit polyclonal anti-Obg peptide primary antibodies (S3 Fig).

### Complementation of MS-H vaccine strain with wild-type *obg* did not restore *ts*^*−*^phenotype

**Fig 5:**
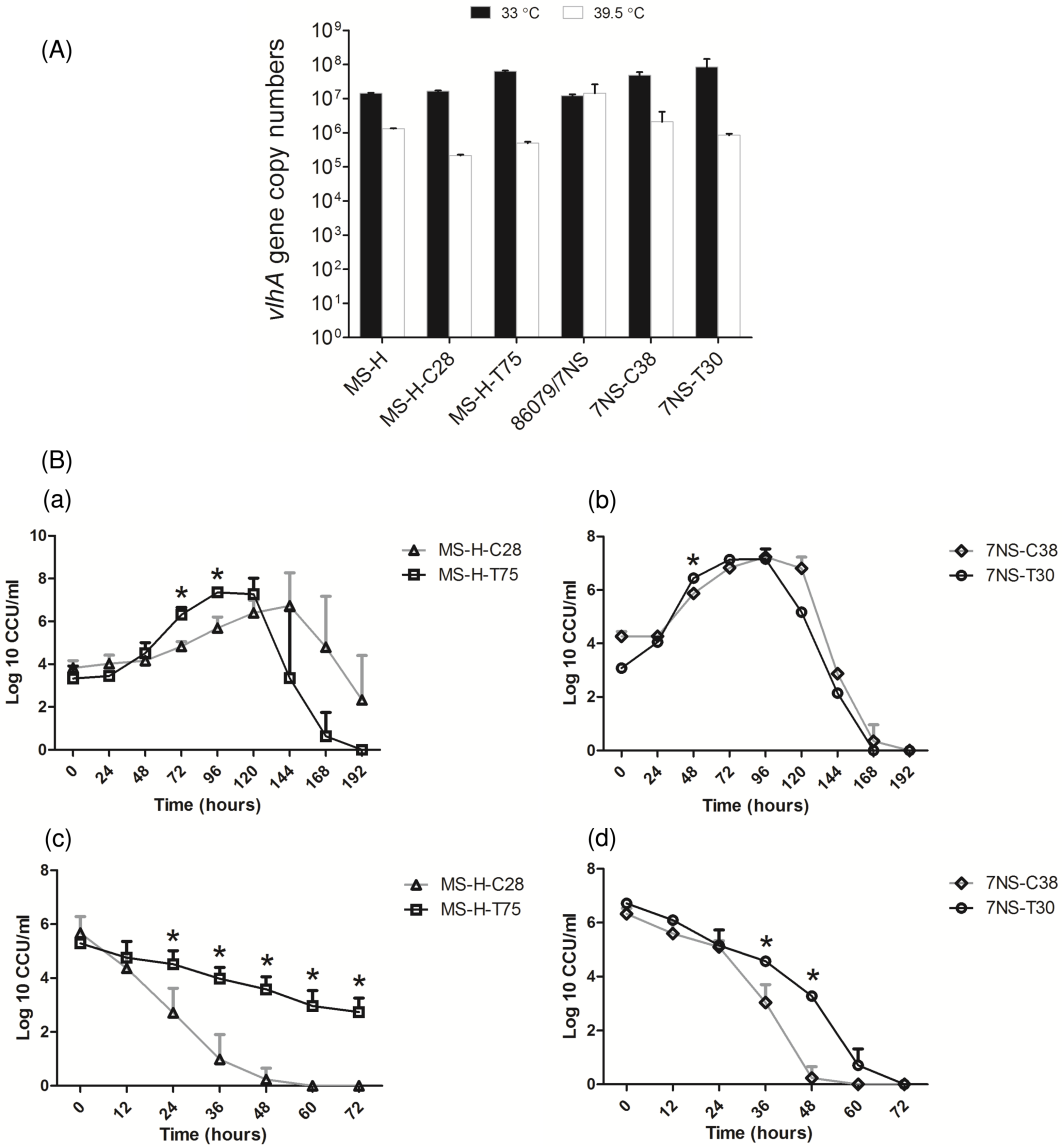
Temperature-sensitive phenotyping, growth curve analysis and loss of viability study of MS-H and 86079/7NS transformants. (A) Using *vlhA*-QPCR as described previously [29], quantification of *vlhA* gene copy number was performed at 33 and 39.5°C for untransformed MS-H (*ts*^+^ control) and 86079/7NS (*ts*^*−*^control), transformed MS-H and 86079/7NS with pMAS-LoriC (clone MS-H-C28 and 7NS-C38, respectively) and pKS-VOTL (clone MS-H-T75 and 7NS-T30, respectively). Growth was lower at non-permissive temperature (39.5°C) than permissive temperature (33°C) for all strains/transformants except 86079/7NS. Data is expressed as mean ± SD of *vlhA* gene copy numbers (n = 3). The 33/39.5°C *vlhA* gene copy number ratios, as established by Shahid et al. [29], indicated MS-H, MS-H-C28, MS-H-T75, 7NS-C38 and 7NS-T30 as *ts*^+^ and 86079/7NS as *ts*^*–*^. (B) Growth curve analysis and loss of viability study of MS-H and 86079/7NS transformants. (a and b) For growth rate comparison, cultures of MS-H-C28 (Δ), MS-H-T75 (ϒ), 7NS-C38 (◊) and 7NS-T30 (○) transformants were grown at 33°C for 192 h. Aliquots were collected at 24 h intervals, and then titrated by incubation at 33°C for two weeks. (c and d) For loss of viability study, cultures of MS-H and 86079/7NS transformants, as indicated in panel a and b, were grown at 39.5°C for 72 h, aliquots collected at 12 h intervals, and then titrated by incubation at 33°C for two weeks. All cultures were grown in MB containing tetracycline (3 μg mL^–1^). Bars indicate standard deviations of log_10_-transformed CCU mL^–1^ values from three independent cultures and "*" indicates significant differences (p < 0.05) using Student's *t*-test.

The numbers of *vlhA* gene copies at 39.5°C were lower in all cultures when compared to those at 33°C for each strain/transformant except vaccine parent strain 86079/7NS (used as *ts*^−^control). The Q-PCR 33/39.5°C *vlhA* gene copy number ratios of MS-H, MS-H-C28 (MS-H transformed with pMAS-LoriC), MS-H-T75 (MS-H transformed with pKS-VOTL), 7NS-C38 (86079/7NS transformed with pMAS-LoriC) and 7NS-T30 (86079/7NS transformed with pKS-VOTL) were 11, 77, 127, 23 and 100, respectively, all indicating a *ts*^+^ phenotype whilst a ratio of 0.8 for 86079/7NS indicating a *ts*^−^phenotype (Fig 5A). Examination of the cultures using conventional microtitration also showed that all cultures, except 86079/7NS, exhibited no growth (change of MB color) at the non-permissive temperature, and hence, were *ts*^+^ (results not shown).

### Growth promoting effect of Obg was only evident in pKS-VOTL transformed MS-H

Growth curve analysis of MS-H-C28 (MS-H transformed with pMAS-LoriC) and MS-H-T75 (MS-H transformed with pKS-VOTL) revealed that MS-H-T75 had significantly faster (p < 0.05) growth rate and shorter doubling/ generation time than MS-H-C28 during exponential phase. The generation times calculated for MS-H-C28 at 48, 72, 96, 120 and 144 h time points during exponential phase (starting from 24 h time point) were 51.6, 17.8, 13.1, 12.2 and 13.3 h per generation, respectively, with an average generation time of 21.6 ± 16.9 h per generation. Similarly, the generation times for MS-H-T75 at 48, 72, 96 and 120 h time points (starting from 24 h time point) were 6.8, 5.0, 5.5 and 7.5 h per generation, respectively, with an average of 6.2 ± 1.2 h per generation (Fig 5B, panel a). Growth curve analysis indicated that 7NS-T30 (86079/7NS transformed with pKS-VOTL) had faster (p < 0.05) growth rate than 7NS-C38 (86079/7NS transformed with pMAS-LoriC). The generation times for 7NS-C38 at 48, 72 and 96 h (starting from 24 h time point) during exponential phase, were 4.5, 5.6 and 7.3 h per generation, respectively, with an average of 5.8 ± 1.4. At similar time points, the generation times for 7NS-T30 were 3.0, 4.7 and 7.0 h per generation with an average of 4.9 ± 2.0 h per generation (Fig 5B, panel b).

Using temperature shift experiment, it was shown that both transformants MS-H-T75 and MS-H-C28 couldn't grow at the non-permissive temperature. However, MS-H-T75 gradually lost its viability at 39.5°C and remained viable at least up to 72 h of incubation at 39.5°C. MS-H-C28 lost viability relatively quickly and couldn't survive after 36 h of incubation at 39.5°C (Fig 5B, panel c). Similarly, 7NS-T30 and 7NS-C38 couldn't grow at 39.5°C. Contrary to MS-H-T75, 7NS-T30 completely lost viability at 72 h of incubation at 39.5°C while 7NS-C38, like MS-H-C28, lost viability after 36 h of incubation (Fig 5B, panel d).

## Discussion

Effect of Obg on growth characteristics has been documented in several bacterial species [6, 16, 36–38]; however, this is the first study examining the role of Obg in any mycoplasma species under experimental conditions so far. A previous study in our group suggested a link between mutation in Obg and *ts* phenotype of *M*. *synoviae* [24]. In the current study, the effect of MS-H *obg* gene SNP (G367A) on temperature sensitivity and growth characteristics of *M*. *synoviae* was further investigated under experimental conditions. The *obg* gene was amplified from vaccine parent strain 86079/7NS, cloned into pKS-VOTL, and successfully transformed into MS-H. Plasmid was shown to be integrated completely into the chromosomal *oriC* and no incidence of homologous recombination at *obg* or any other locus in the MS-H genome was observed. The ~ 3.9 kb *vlhA*-*obg* transcript (transcribed from integrated plasmid), detected by Northern blot hybridisation, may have comprised the downstream region of the plasmid pKS-VOTL (*obg*-containing plasmid) as there was no transcription termination signal cloned for *M*. *synoviae obg*. Two bands of ~ 1.4 and 1.2 kb detected by *vlhA*-*obg* and *obg* probes in total RNA of MS-H pKS-VOTL transformants, indicated abundance of chromosomal *obg* transcript(s). It is likely that the expression of wild-type *obg* may have enhanced the transcription of chromosomal *obg* in the transformed cells by unknown mechanism(s) which might involve either the upregulation or enhanced activity of transcription factor complexes, or the inability of transcription regulators, e.g., miRNA-like RNAs [39] to cope with this overexpression.

The results presented here suggest that the expression of endogenous Obg was less abundant in untransformed MS-H and MS-H transformed with pMAS-LoriC (non-*obg* plasmid) as compared to untransformed 86079/7NS and its pMAS-LoriC transformant. The abundance of endogenous Obg protein in 86079/7NS as compared to MS-H was more evident in their transformants with wild-type *obg*-containing plasmid (pKS-VOTL). Interestingly, MS-H5, one of the *ts*^*−*^MS-H reisolates with back mutation at position 123 of Obg [24, 29], exhibited detectable levels of endogenous Obg beyond that of MS-H. Previous studies have reported that missense mutations might accelerate the degradation of the mutant protein by proteases and hence reduce the concentration of the mutant gene product [40–42]. It has been shown that *ts*^+^ *E*. *coli* mutants possess proteins with a rapid turnover, e.g. a *ts*^+^ RNA polymerase was found hydrolysed rapidly, especially at the nonpermissive temperature [43]. Therefore, it may be possible that reduced level of MS-H Obg is due to Gly123Arg substitution, resulting in mutant protein instability and rapid degradation. It may be speculated that mutant protein instability and lower concentration may not hinder its biological functions at a permissive temperature but structural defect and concentration lower than a certain critical level may block its biological functions at higher temperature.

In this study, effect of *obg* complementation on growth characteristics of MS-H was investigated. Subcultures of MS-H pKS-VOTL transformant colonies, grown in MB containing tetracycline, exhibited faster growth rates (observed by rapid acidification of the broth medium) as compared to MS-H pMAS-LoriC transformant colonies at permissive temperatures of 33 and 37°C. Growth curve analysis of MS-H and 86079/7NS transformants (with both plasmids) showed clear growth promoting effect of Obg only in MS-H, suggesting that MS-H Obg with Gly123Arg substitution is functionally impaired. When cultures of MS-H and 86079/7NS transformants were titrated, and shifted to 33°C following incubation at nonpermissive temperature, pKS-VOTL transformants of both MS-H and 86079/7NS maintained viability for longer time than pMAS-LoriC transformants. The ability to maintain viability, despite being growth arrested at the nonpermissive temperature (39.5°C), suggests at least partial biological function of the wild-type Obg expressed from pKS-VOTL.

Obg has binding site for ppGpp (guanosine 5′-diphosphate 3′-diphosphate) which is abundantly synthesized under stress conditions [13] and is responsible for the readjustment of gene expression according to the endured conditions [44]. Obg might hydrolyze pppGpp into ppGpp and regulate pppGpp/ppGpp ratio which has been linked to replication fork stability and cell survival [7, 12, 44–46]. Recently, Obg has been proposed as a checkpoint protein in the assembly of 50S ribosome subunit, sensing the energy status of the cell via (p)ppGpp levels and linking ribosome assembly to other global growth control pathways [47]. In present study, MS-H complementation with wild-type *obg* could not restore *ts* phenotype, identical to that of the wild-type strain (86079/7NS), suggesting that contribution of Obg mutation towards MS-H temperature sensitivity phenotype may involve a complex mechanism. Furthermore, complete genome sequencing of MS-H and 86079/7NS has revealed that MS-H harbours several other mutations, some of which are mis-sense mutations in other essential genes (unpublished data). However, it is not yet clear if those mutation(s), either independently or in conjunction with *obg* gene SNP, could influence the temperature sensitivity and/or attenuation of MS-H.

It is possible that Obg overexpression, as observed in this study, exert an inhibitory effect on some unknown factor(s), required only under stress conditions (e.g., high temperature), and that the overexpression of Obg may have abolished *in vivo* function of unknown factor(s). In *S*. *coelicolor* and *S*. *griseus*, bacteria undergoing morphological development, overexpression of Obg had no effect on normal vegetative growth but inhibited aerial mycelium and thus spore formation [38, 48]. Aerial mycelium followed by spore development is initiated under adverse environmental conditions and the level of Obg decreased dramatically just after the onset of aerial mycelium formation, and essentially no Obg was detected in cells during spore formation. Obg seems to mediate transition from vegetative to non-vegetative growth by sensing the intracellular GTP/GDP levels [38, 48]. Obg-GTP form normally promotes vegetative growth while Obg-GDP stimulates aerial mycelium and spore formation. It is known that under stress conditions the level of GTP expression is decreased and therefore it is speculated that stress may induce a direct signal for Obg down regulation. It has been proposed that Obg overexpression suppress aerial mycelium and spore formation, and this suppression results from the presence of the excess Obg bound to GTP under conditions when Obg would normally bound to GDP [12, 38].

Depletion or overexpression of Obg lead to growth defects in *E*. *coli* [14, 49, 50], *B*. *subtilis* [6, 51], *S*. *coelicolor* [38] and *C*. *crescentus* [16]. Changing the level of Obg expression has significant effects on cell morphology, nucleoid appearance, chromosome segregation and regulation of chromosomal functions [12]. Consistent with these observations, overexpression of wild-type Obg in MS-H (transformant MS-H-T75), and 86079/7NS (transformant 7NS-T30), had no deleterious effect on growth at permissive temperature (33°C) but might have growth inhibitory effect at nonpermissive temperature (39.5°C). In *M*. *synoviae*, Obg concentration might also be regulated in response to transition from favorable to unfavorable growth conditions/temperatures; however, further studies using *obg* native promoter or other inducible promoters are required to prove this hypothesis.

It was puzzling that transformation of the wild-type parent strain 86079/7NS with the wild-type *obg*-bearing construct and no-*obg* bearing vector resulted in temperature sensitivity phenotype (clones 7NS-T30 and 7NS-C38 respectively) in this study. Temperature sensitivity of 7NS-T30 might be explained by the overexpression of Obg (as discussed above for MS-H-T75) while temperature sensitivity of 7NS-C38 was unexpected. The real cause of this result could not be found in the current study. It is perhaps worthwhile examining if spontaneous mutations in plasmid-encoded proteins (e.g., antibiotic resistance proteins, replication initiation proteins) may have occurred causing replication abnormalities in the host only at the high or nonpermissive temperature. Mutations in replication associated proteins encoded by plasmids can eliminate, reduce or even stimulate temperature sensitivity to host bacterial strains [52–54]. A number of mutations in TrfA, the only plasmid-encoded protein required for initiation of replication of the broad-host-range mini-RK2 plasmid, have been shown to induce temperature sensitivity in *E*. *coli* and *Pseudomonas aeruginosa* [54] while intragenic second-site copy up (*cop*) mutations of *trfA* eliminated or strongly reduced temperature sensitivity in *P*. *aeruginosa* [53]. Future studies should also include transforming 86079/7NS or any other *ts*^*−*^wild-type *M*. *synoviae* field strain with a construct containing mutant (MS-H type) *obg* gene, or generate such a mutant using reverse genetic techniques, and to examine its temperature sensitivity phenotype.

## Conclusion

In conclusion, complementation of *ts*^+^ MS-H vaccine with wild-type *obg* could not restore *ts*^*−*^phenotype; however, wild-type *obg* expression had growth promoting effect at the permissive temperature whilst it improved the ability to survive at the nonpermissive temperature. Furthermore, with the development of first expression system *in M*. *synoviae*, development of the recombinant MS-H vaccine may also be feasible. To establish the role of Obg mutations in temperature sensitivity and/or attenuation of MS-H vaccine, further work is essential, either by expressing *obg* under its natural or a regulable (inducible) promoter allowing for the examination of Obg on *M*. *synoviae* growth at expression level lower than that observed in this study.

## Supporting information

S1 Fig Northern analysis of total RNA (~ 25 μg/lane) from *M*. *synoviae* strain MS-H, MS-H-C28, MS-H-T75, MS-H-T78 and MS-H-T90. Northern blots were hybridised with DIG-labeled *vlhA*-*obg* probe (spanning the joining site of *vlhA* promoter with *obg* CDS), *obg* specific probe and *vlhA* coding sequence specific probe. Arrowheads indicate the location of the specific bands identified by probes described above. The location of bands for the DIG-labeled RNA molecular weight marker RNA I (Roche) is indicated on the left side of all northern blots.
(TIF)

S2 Fig Analysis of Obg expression in *E*. *coli* and *M*. *synoviae* using SDS-PAGE and immunoblotting. (A) SDS-PAGE of *E*. *coli* lysates from noninduced culture, induced culture, supernatant from induced culture, and affinity purified Obg-MBP. Arrows indicate location of the recombinant *M*. *synoviae* Obg and MBP proteins in *E*. *coli*, respectively. (B) Immunostaining of *M*. *synoviae* strain MS-H transformed with pMAS-LoriC (clone MS-H-C28) and pKS-VOTL (clones MS-H-T75, MS-H-T78 and MS-H-T90) using polyclonal chicken anti-Obg-MBP serum. Several nonspecific bands ranging from 80–100 kDa were detected but not a band of ~ 50 kDa in *M*. *synoviae* strains. Affinity purified Obg-MBP was used as positive control. Location of Obg-MBP and MBP is indicated on the right.
(TIF)

S3 Fig Comparison of Obg expression in MS-H and 86079/7NS. Immunostaining at two different concentrations of whole cell proteins (i.e. 1/3 and 2/3 of that loaded onto SDS-PAGE as shown in Fig 4E) of each strain/transformant probed with polyclonal rabbit serum against *M*. *synoviae* Obg_1 peptide. MW, protein marker (Precision Plus Protein, Dual Color, Bio-Rad).
(TIF)

S1 Table Oligonucleotides used in this study for PCR amplifications and probe synthesis. (DOCX)

S1 File Splicing by overlap extension PCR. (DOCX)

S2 File Expression of *M*. *synoviae* Obg in *E*. *coli* and production of Obg-MBP antiserum in chickens. (DOCX)
