# Supplementary figures and images for: Complementation of the Mycoplasma synoviae MS-H vaccine strain with wild-type obg influencing its growth characteristics
Source: PLoS One. 2018 Mar 28;13(3):e0194528. doi: 10.1371/journal.pone.0194528 (PMC5874028; doi:10.1371/journal.pone.0194528)

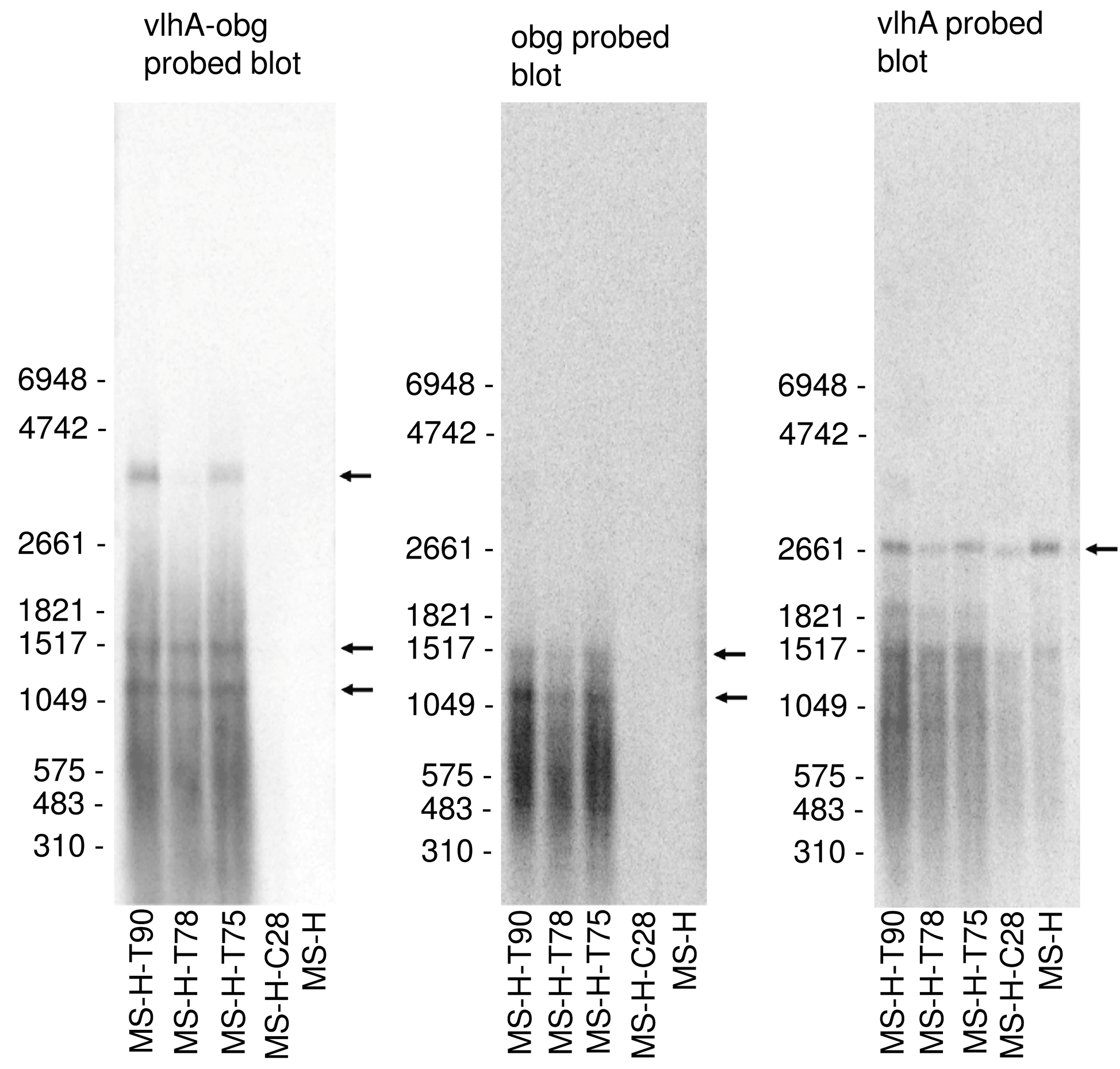

Supplement: S1 Fig — Northern blots were hybridised with DIG-labeled vlhA-obg probe (spanning the joining site of vlhA promoter with obg CDS), obg specific probe and vlhA coding sequence specific probe. Arrowheads indicate the location of the specific bands identified by probes described above. The location of bands for the DIG-labeled RNA molecular weight marker RNA I (Roche) is indicated on the left side of all northern blots. (TIF) [file pone.0194528.s001.tif]

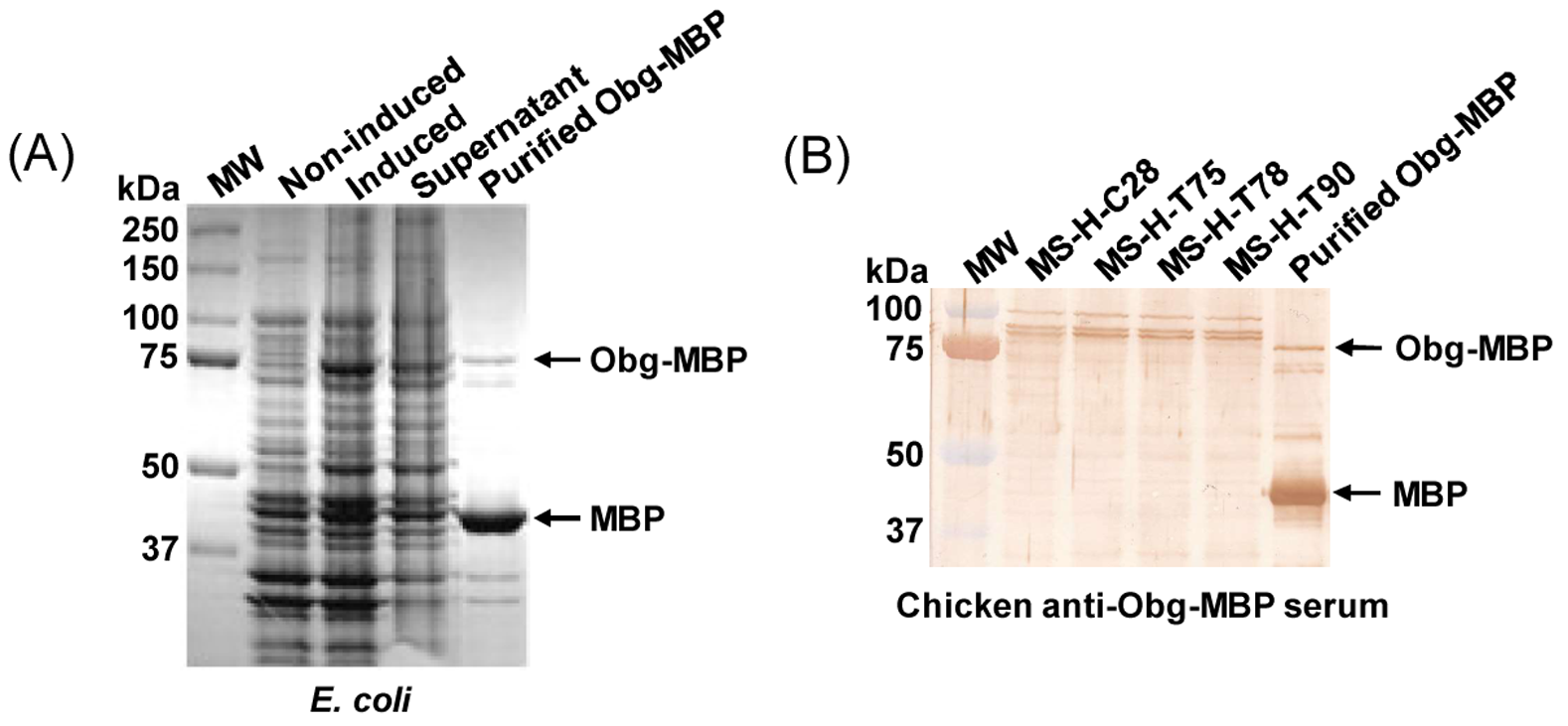

Supplement: S2 Fig — (A) SDS-PAGE of E. coli lysates from noninduced culture, induced culture, supernatant from induced culture, and affinity purified Obg-MBP. Arrows indicate location of the recombinant M. synoviae Obg and MBP proteins in E. coli, respectively. (B) Immunostaining of M. synoviae strain MS-H transformed with pMAS-LoriC (clone MS-H-C28) and pKS-VOTL (clones MS-H-T75, MS-H-T78 and MS-H-T90) using polyclonal chicken anti-Obg-MBP serum. Several nonspecific bands ranging from 80–100 kDa were detected but not a band of ~ 50 kDa in M. synoviae strains. Affinity purified Obg-MBP was used as positive control. Location of Obg-MBP and MBP is indicated on the right. (TIF) [file pone.0194528.s002.tif]

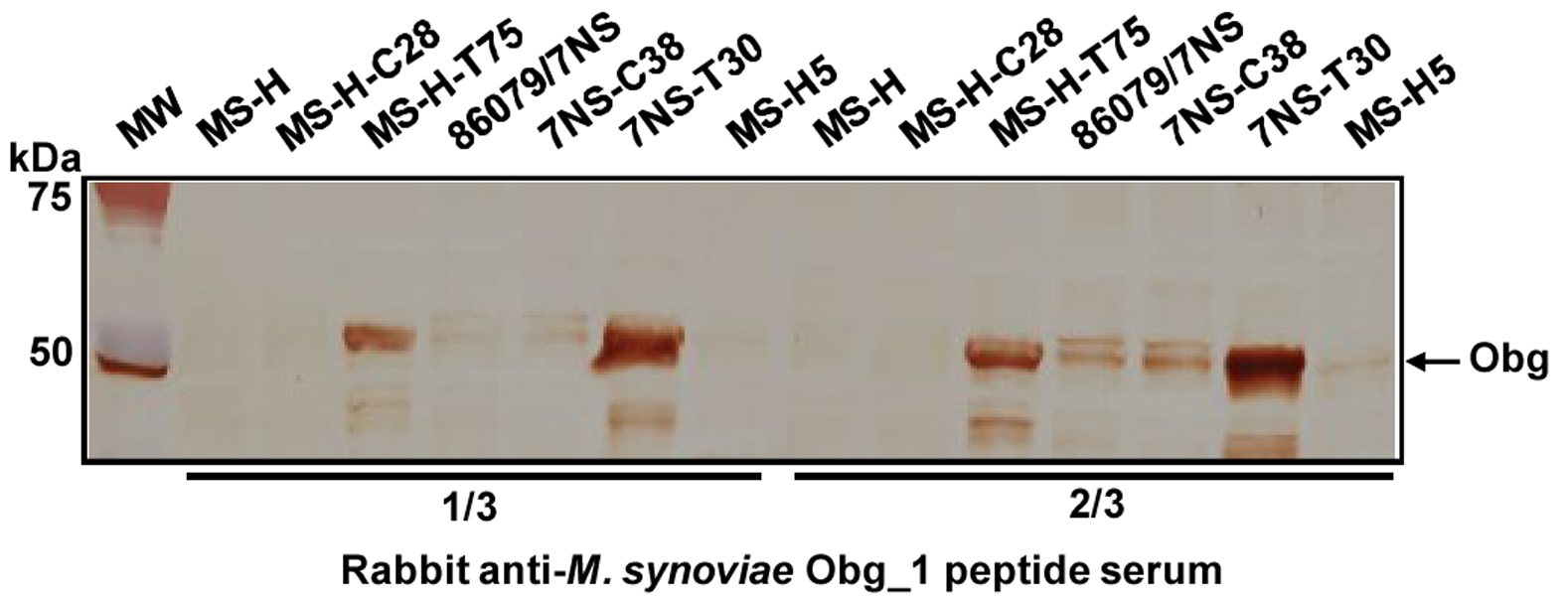

Supplement: S3 Fig — Immunostaining at two different concentrations of whole cell proteins (i.e. 1/3 and 2/3 of that loaded onto SDS-PAGE as shown in Fig 4E) of each strain/transformant probed with polyclonal rabbit serum against M. synoviae Obg_1 peptide. MW, protein marker (Precision Plus Protein, Dual Color, Bio-Rad). (TIF) [file pone.0194528.s003.tif]
