# Supplementary material for: Complementation of the Mycoplasma synoviae MS-H vaccine strain with wild-type obg influencing its growth characteristics
Source: PLoS One. 2018 Mar 28;13(3):e0194528. doi: 10.1371/journal.pone.0194528 (PMC5874028; doi:10.1371/journal.pone.0194528)
Supplement: S1 Table — (DOCX) [file pone.0194528.s004.docx]

S1 Table. ^a^Oligonucleotides used in this study for PCR amplifications and probe synthesis.

| **Primer name** | **Sequence (5′-3′)** | **Amplified region/usage** | **Size of amplified fragment (bp)** |
| --- | --- | --- | --- |
| oriC-F2  oriC-R1 | AATTGGTCGTGACTTTAACATG  CATTATTGTGTTGATCGAATTCTAC | Synthesis of *oriC* probe | 668 |
| oriC-F1 | TAGCGTTTCTATAGTTGACGA | Sequencing | NA |
| oriC-R2 | GTAAATGTTGTTAAATTCGCCAG | Sequencing | NA |
| malE-F | GGTCGTCAGACTGTCGATGAAGCC | Sequencing | NA |
| tetM-F1  tetM-R1 | GCAGTTATGGAAGGGATACG  TTCTTGAATACACCGAGCAG | Tetracycline resistance gene *tetM* and synthesis of *tetM* probe | 340 |
| vlhA-extF  vlhA-intR | TAATAGCTTGCCTTAAAGCG  CTTGATCTATAAACTTTGCCATAAATAAATCCTTTTATTTTACGCA | *vlhA* promoter region | 662 |
| obg-intF  obg-extR | TGCGTAAAATAAAAGGATTTATTTATGGCAAAGTTTATAGATCAAG  AAGTTAAATCACCTGTTGGC | *obg* complete CDS | 1323 |
| vlhA-extF  obg-extR | TAATAGCTTGCCTTAAAGCG  AAGTTAAATCACCTGTTGGC | *vlhA* promoter region plus *obg* complete CDS in SOE PCR | 1985 |
| obg-*Bam*HI  obg-*Hin*dIII | TCATGCTCGGATCCGGTAAAGGAGGTGATGG  TTATAAAGCTTCCTCCCATTCAAATTTATG | Amplification of partial *obg* | 1247 |
| C52-5F  obg-Ri2 | AATTTATTTTTGCTAAAATT  AGAGGTTTTAAATTTATTATTTCC | Synthesis of *vlhA*-*obg* probe spanning ligation site of *vlhA* promoter region with *obg* CDS | 637 |
| Link-F  MS-Cons-R | TACTATTAGCAGCTAGTGC  AGTAACCGATCCGCTTAAT | Synthesis of *vlhA* specific probe | 380 |
| obg-F  obg-R | GTTGATAAAGGTGGACCAG  TTAGTGCAGATATCTCAATG | Synthesis of *obg* specific probe | 841 |

^a^ All oligonucleotides were synthesized by GeneWorks, Pty. Ltd., Australia; NA, not applicable.

Restriction sites are underlined
