# Supplementary material for: Complementation of the Mycoplasma synoviae MS-H vaccine strain with wild-type obg influencing its growth characteristics
Source: PLoS One. 2018 Mar 28;13(3):e0194528. doi: 10.1371/journal.pone.0194528 (PMC5874028; doi:10.1371/journal.pone.0194528)
Supplement: S1 File — (DOCX) [file pone.0194528.s005.docx]

### Splicing by overlap extension PCR

As *obg* gene regulatory elements have not been characterised in mycoplasmas, *vlhA* gene promoter region was joined to *obg* CDS, using splicing by PCR-driven overlap extension as described before [[1](#_ENREF_1)] with modifications. Briefly, amplification of *vlhA* promoter region was conducted in a 20 µL PCR mixture containing 0.4 µL each of 25 µM vlhA-extF and vlhA-intR oligonucleotides (S1 Table), 4 µL of 5× Phusion HF buffer, 3% dimethyl sulfoxide (DMSO), 3.2 µL dNTP mixture containing 1.25 mM each of dATP, dGTP, dCTP and dTTP, 1 U of Phusion^®^ High-Fidelity DNA Polymerase (New England Biolabs, Wilbury Way, Hitchin, England) and 3 µL of genomic DNA from *M. synoviae* strain 86079/7NS. Amplification conditions consisted of one cycle of 30 sec at 98 °C as initial denaturation of DNA followed by 35 cycles of 98 °C for 10 sec, 56 °C for 30 sec and 72 °C for 30 sec followed by final extension of 5 min at 72 °C. *obg* CDS was amplified using the same reagents and conditions as for *vlhA* promoter PCR except extension step of 50 sec at 72 °C during 35 cycles of amplification. Amplicons from these two PCRs had overlapping nucleotide sequences which were extended in the overlap extension PCR using the oligonuclotides vlhA-extF and obg-extR (S1 Table). Overlap extension PCR was performed in a 25 µl reaction mixture using 1U of Platinum^®^ Taq DNA Polymerase high fidelity (Invitrogen, Mount Waverley, Victoria, Australia), 2.5 µL each of *vlhA* promoter (~ 50 ng µL^–1^) and *obg* CDS (~ 100 ng µL^–1^) amplicons as template, 2.5 µl of 10× high fidelity PCR buffer, 1 µL each of 25 µM vlhA-extF and obg-extR oligonuclotides, 1 µL of 50 mM MgSO_4_, 4 µL of dNTP mixture containing 1.25 mM each of dATP, dGTP, dCTP and dTTP and 10.3 µL of nuclease free water. PCR conditions consisted of an initial denaturation at 94 °C for 2 min followed by 40 cycles of 94 °C for 30 sec, 55 °C for 30 sec and 68 °C for 2.5 min. Amplicons of expected size were confirmed by agarose gel electrophoresis (Fig 1C).

**References**

1. Heckman KL, Pease LR. Gene splicing and mutagenesis by PCR-driven overlap extension. Nat Protoc. 2007;2(4):924-32.
