# Supplementary material for: Complementation of the Mycoplasma synoviae MS-H vaccine strain with wild-type obg influencing its growth characteristics
Source: PLoS One. 2018 Mar 28;13(3):e0194528. doi: 10.1371/journal.pone.0194528 (PMC5874028; doi:10.1371/journal.pone.0194528)
Supplement: S2 File — (DOCX) [file pone.0194528.s006.docx]

### Expression of *M. synoviae* Obg in *E. coli* and production of Obg-MBP antiserum in chickens

Using primers obg-*Bam*HI and obg-*Hin*dIII (S1 Table), partial *obg* CDS from *M. synoviae* strain 86079/7NS was PCR amplified, and then cloned into pGEM^®^-T Easy vector, according to manufacturer's instructions (Promega, Alexandria, New South Wales, Australia), to generate pGEM-T-obg. Digestion of pGEM-T-obg with *Bam*HI and *Hin*dIII (New England Biolabs), as per manufacturer’s recommendations, released an *ob*g fragment which was ligated into *Bam*HI and *Hin*dIII restricted pMAL-p2 expression vector (New England Biolabs) to produce pMAL-obg. The sequence of the insert in pMAL-obg plasmid was verified by DNA sequencing using malE-F and M13-F primers. *E. coli* was transformed with pMAL-obg and ampicillin resistant colonies, exhibiting IPTG (isopropylthiogalactoside) inducible expression of ~ 77 kDa Obg-MBP fusion protein, were selected for expression and purification of fusion protein using amylose resin beads (New England Biolabs) as per manufacturer’s recommendations. Purified Obg-MBP was subjected to SDS-PAGE and a band corresponding to fusion protein was excised, emulsified by sonication with Montanide ISA 71 VG (Seppic Inc., Puteaux, France) adjuvant, and used to immunise three 14-day old SPF chickens by intramuscular injections. Chickens were boosted twice with similar injections and bled after 14 days following each injection. Sera were tested for anti-Obg antibodies by immunoblotting as described in Materials and methods in the main manuscript.
